# A Comparison of Scent Marking between a Monogamous and Promiscuous Species of *Peromyscus*: Pair Bonded Males Do Not Advertise to Novel Females

**DOI:** 10.1371/journal.pone.0032002

**Published:** 2012-02-29

**Authors:** Elizabeth A. Becker, Sarah Petruno, Catherine A. Marler

**Affiliations:** 1 Department of Psychology, University of Wisconsin–Madison, Madison, Wisconsin, United States of America; 2 Department of Zoology, University of Wisconsin–Madison, Madison, Wisconsin, United States of America; 3 Division of Biological Sciences, University of California San Diego, La Jolla, California, United States of America; University of Western Ontario, Canada

## Abstract

Scent marking can provide behavioral and physiological information including territory ownership and mate advertisement. It is unknown how mating status and pair cohabitation influence marking by males from different social systems. We compared the highly territorial and monogamous California mouse (*Peromyscus californicus*) to the less territorial and promiscuous white-footed mouse (*P. leucopus*). Single and mated males of both species were assigned to one of the following arenas lined with filter paper: control (unscented arena), male scented (previously scent-marked by a male conspecific), or females present (containing females in small cages). As expected, the territorial *P. californicus* scent marked and overmarked an unfamiliar male conspecific's scent marks more frequently than *P. leucopus*. Species differences in responses to novel females were also found based on mating status. The presence of unfamiliar females failed to induce changes in scent marking in pair bonded *P. californicus* even though virgin males increased marking behavior. Pair bonding appears to reduce male advertisement for novel females. This is in contrast to *P. leucopus* males that continue to advertise regardless of mating status. Our data suggest that communication through scent-marking can diverge significantly between species based on mating system and that there are physiological mechanisms that can inhibit responsiveness of males to female cues.

## Introduction

Odorant communication has been adopted by a wide range of vertebrate and invertebrate species as a relatively inexpensive mode of communication compared to other types of communication. Among mammals, a wealth of research suggests that scent marking is used to signal receptivity [1] and attract potential mates [2]–[4], as well as advertise territory ownership [5], [6], territorial boundaries [7], competitive ability [5], [8]–[10] and dominance (review by [11]). Chemical signals have a variety of other functions as exemplified by those observed in invertebrates such as food source and location identification [12]–[13] and synchronization of reproductive behavior [14]. Modulation of chemical signals may be limited compared to other modes of communication such as acoustic signals, but nonetheless we expect some modulation to occur in response to environmental conditions. For example, there may be both social and survivorship (predation risk) [11] consequences for scent-marking marking behavior. Our goal was to examine how the social system influences scent-marking behavior in response to conspecifics from both a territorial and sexual perspective. We wanted to examine how males respond to the scent marks of an unfamiliar male, as well as to the presence of unfamiliar females. Of particular interest was the effect of pair bonding in a monogamous species on scent mark signaling.

One way to test for the function of scent marking is to compare how scent marking patterns differ before and after pair bonding in a monogamous species and then compare that with species that do not form pair bonds. Few studies compare species with different mating systems (but see [15], [16]) or levels of territoriality. We studied two closely related species with different mating systems and levels of territoriality. The strictly monogamous [17] California mouse (*Peromyscus californicus*) is highly aggressive (e.g. [18]–[23]) and males display year-round territoriality [24]. Moreover, males exhibit an increase in post-encounter testosterone (with no change in corticosterone) after and aggressive encounter that increases future ability to win aggressive encounters with other males [18], [19], [25]–[27]. In contrast, the closely related and promiscuous white-footed mouse (*Peromyscus leucopus*) is less aggressive [28], [29], less territorial [30], [28], and promiscuous [31]. Males typically have home ranges that overlap those of several females [32] and will adopt a wandering strategy [33], [34]to find mates. Males display no increase in testosterone after an aggressive encounter and do not exhibit a significantly increased ability to win after repeated encounters [29], [23]. *P. californicus* males also tend to attack on the back and flank areas, whereas *P. leucopus* males tend to attack on the snout and face [29] *P. californicus* males may therefore show more “offensive” aggression while P. leucopus males may show more “defensive” aggression based on definitions established by [35] We tested the following predictions related to species differences in behavior and pair bond status.

On a species level, with no exposure to conspecific stimuli, if the primary function of scent marking is to mark the boundaries of their territory in order to dissuade intrusion from other males, then we predicted that the territorial *P. californicus* would scent mark at higher frequencies, especially around the perimeter [6], as compared to *P. leucopus*. Males that are stressed, particularly subordinate males, also urinate around the perimeter, but do so in few areas and typically produce a limited number of “pools” of urine [36]. Similarly, we expected the highly aggressive *P. californicus* to scent mark and overmark more than *P. leucopus* if the function is self-advertisement of competitive ability [5], [8], [10], [37]. When exposed to conspecific stimuli, we had different predictions for each species based on mating status (cohabitation with mate versus group housed with other males). We predicted that pair-housing (pair bonding in the case of *P.californicus*) would increase marking behavior in response to a male conspecific scent because of the male's investment in the female and offspring. In *P. leucopus,* however, we predicted less of a response to a male's scent due to less direct competition between males because of wandering to find mates. We predicted that pair bonding in strictly monogamous males would decrease their response to unfamiliar females. In contrast we predicted that males of the promiscuous *P. leucopus* would respond to unfamiliar females with a greater frequency of scent marking if the primary focus of scent marking is mate advertisement [2]–[4].

This is one of the first studies to examine the effect of pair bonding on scent marking behavior and addresses how the function of scent marking may differ between and within species based on the social context (territory establishment, competition, mating) in closely related species.

## Materials and Methods

### Subjects

We used 106 randomly selected male *P. californicus* (47; 24 pair-housed, 23 group-housed) and *P. leucopus* (59; 35 pair-housed, 24 group-housed) mice, ranging in age from six to twelve months, reared in our laboratory colonies at the University of Wisconsin, Madison. Animals were maintained in accordance with the National Institute of Health *Guide for the Care and Use of Laboratory Animals*. Animal treatment and research protocols were approved by the University of Wisconsin, Madison College of Letters and Sciences Institutional Animal Care and Use Committee (IACUC); L0021-0-03-10. During the standard housing phase, focal males were housed in cages (48.3 cm×26.7 cm×15.6 cm) with either a mate or same-sex conspecifics, as well as food and water available *ad libitum*. During the testing phase, individuals were placed in a clean glass aquarium (60×30×30 cm) lined with filter paper (Fisher Brand; Qualitative P8; flow rate: fast). The animals were kept under a 14L∶10D hour light cycle. We conducted behavioral tests under dim red light 30 min after the initiation of the dark phase. The filter paper was immediately examined under ultraviolet light (20 W) to illuminate urine marks and scent marks were measured as described below.

### Scent marking manipulations and urine mark observations

Scent marking tests began by placing the focal male into the filter paper-lined aquarium. Pair-housed and group-housed males were randomly assigned to one of three social contexts; control, male scented, and females present. Individuals in the control context (*P. californicus* = 20, *P. leucopus* = 29) experienced a clean, previously unmarked arena, whereas individuals in the male-scented context (*P. californicus* = 17, *P. leucopus* = 17) experienced an arena with filter paper previously scent marked by an unfamiliar male conspecific. In the ‘females present’ social context (*P. californicus* = 10, *P. leucopus* = 13), individuals experienced an arena containing three unfamiliar females in small boxes (11.43×7.62×9.52 cm) with a wood bottom and three sides covered in ½ cm^2^ wire screen. The boxes were placed one inch apart in the center of the cage, which approximately four inches of space between the boxes and the walls of the arena on all sides. These boxes allowed males to see, hear, smell and deposit urine around the females, while keeping the arena free of female urine. Because males scent mark more to estrus than non-estrus females in a non-monogamous vole species [38], we selected three females from three different pairs of parents to increase the likelihood that at least one of them was in estrus. It is unlikely that females cycle together because births occur daily within our colony. Once placed in the aquarium, a male was allowed to move about and mark freely for 30 min, at which time the male was removed and returned to his home cage.

After scent marking, all urine marks were immediately visualized with a UV light and traced in pencil to be scored later by an observer blind to the test treatments. Using a grid overlay of ½ cm^2^, we scored surface area, total number of marks and overmarks and distribution of marks (perimeter versus center of the arena). Surface area was calculated by adopting a commonly used approach which is to count the number of grid boxes with scent marks in them and dividing by the total number of boxes e.g. [39]. To measure distribution of urine marks, a small rectangle (5 cm from each outer wall) was drawn on the grid. Urine marks deposited inside the rectangle were counted as center marks whereas urine marks deposited outside of the rectangle were considered perimeter marks.

### Data Quantification and Statistical Analysis

Data were analyzed using analysis of variance (ANOVA) followed by independent samples T-testing for comparisons between groups. Tests of specific *a priori* hypotheses were conducted using Bonferroni adjusted alpha levels. Because of methodological differences, comparisons between responses to male scent marked and female's present were not made, instead each group was independently compare to the control for that species. Our species comparisons were limited to main effects and species by social context interactions We excluded further between-species analyses (three-way interactions between species, social context and mating status) because sample sizes are too small for the number of corrections needed. All statistical analyses were conducted using the computer program SPSS (version 18.0, SPSS, Inc., Chicago, IL).

## Results

We found no main effect of species on surface area scent-marked (*P. californicus* or *P. leucopus*) (F(1,105) = 1.738, *p* = 0.19), mating status (paired or group-housed) (F(1,105) = 0.582, *p* = 0.45) or social context (control, male scented, or females present) (F(1,105) = 0.085, *p* = 0.92). Because there was no difference in estimated surface area between the species we statistically compared the two species.

### Total number of scent marks

As predicted, there was a significant main effect of species on total number of marks deposited. The territorial *P. californicus* marked more (Mean = 227.72, SE = 35.45), than did *P. leucopus* (Mean = 127.66, SE = 17.96, *t*(105) = 2.670, *p* = 0.009). This difference remained when surface area was used as a covariate (F(1,105) = 4.889, *p* = 0.029).

**Figure 1:**
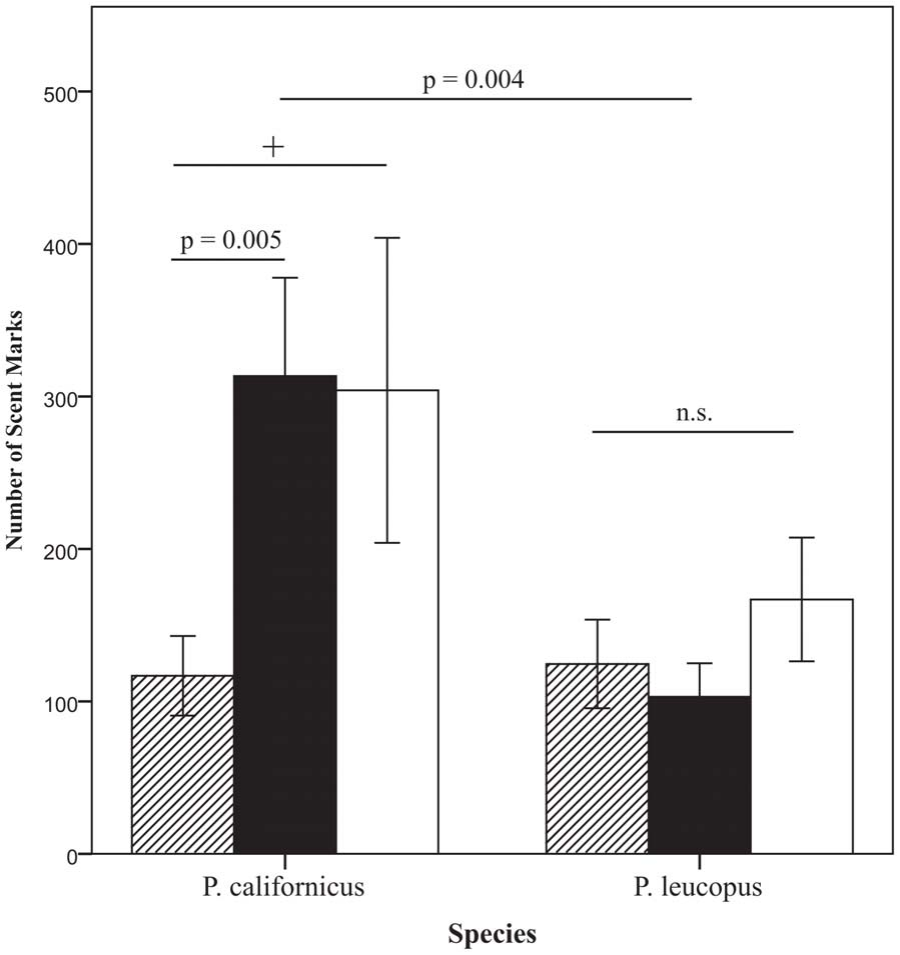
Species differences in total number of scent marks deposited by focal males (Mean ± SE) in response to various social stimuli; control context (hatched bars), male context (black bars), female context (open bars). The+represents a non-significant trend; *p* = 0.025.

Our analyses also revealed a significant two-way interaction between species and social context (F(1,105) = 4.019, *p* = 0.021) (Figure 1). Tests of the *a priori* hypotheses were conducted using Bonferonni adjusted levels of 0.007 per test (0.05/7). Results for all pairwise comparisons are as follows. Male *P. californicus* (Mean = 116.84, SE = 26.11) and *P. leucopus* (Mean = 124.55, SE = 29.04) did not differ in total marks deposited in the control context (t(1, 49) = −0.188, *p* = 0.852), or with unfamiliar females present (*t*(1, 23) = 1.390, *p* = 0.179). However, male *P. californicus* marked more in response to a male conspecific (Mean = 313.35, SE = 64.44) than *P. leucopus* (Mean = 103.00, SE = 22.03, *t*(1, 32) = 3.089, *p* = 0.004).

Results of within-species comparisons (collapsed across mating status) indicated that *P. californicus* marked more in response to the male-marked arena (Mean = 313.35, SE = 64.44) than in the control arena (Mean = 116.79, SE = 26.12, *t*(1, 35) = −2.99, *p* = 0.005). There was a non-significant trend (due to large number of corrections) for male *P. californicus* to exhibit greater marking behavior in response to females (Mean = 304.00, SE = 100.00) than in the control arena (Mean = 166.85, SE = 26.12, *t*(1, 23) = −1.39, *p* = 0.025). *P. leucopus* showed no difference from control levels in marking behavior in any of the social contexts (male stimuli; *t*(1,45) = 0.518, *p* = 0.607; female stimuli; *t*(1,29) = −1.471, *p* = 0.152). Because no differences in *P. leucopus* were found, we limited further analyses to *P. californicus*.

**Figure 2:**
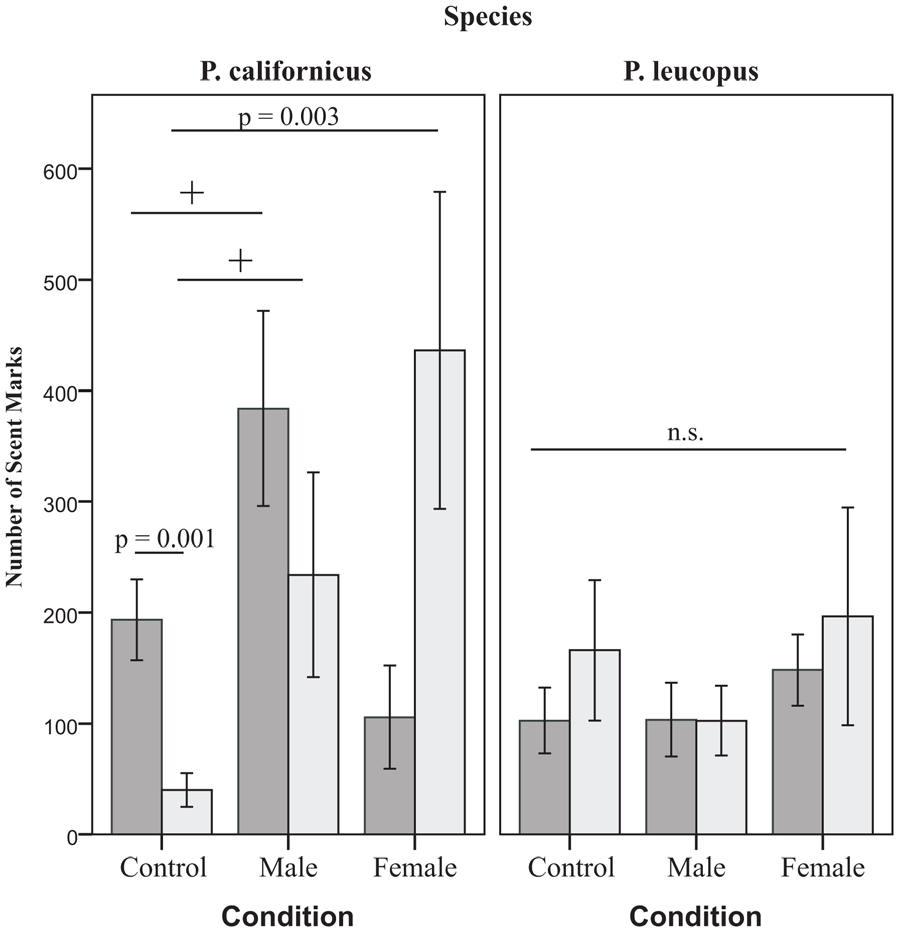
Species differences and mating status influence total number of scent marks deposited by focal males (Mean ± SE) in response to various social stimuli. Dark gray bars represent pair-housed males, while light gray bars represent group housed (virgin) males. The+denotes non-significant trends for response to male stimuli in pair bonded and single *P. californicus*; *p* = 0.05 and *p* = 0.03 respectively.

A significant two-way interaction (F(5,47) = 4.085, *p* = 0.004) (Figure 2) between social context (control, male scented, or females present) and mating status in *P. californicus* was found. Pairwise within species *a priori* hypotheses were tested using Bonferonni adjusted levels of 0.007 per test (0.05/7). In the control arena, mated (bonded, in the case of *P. californicus*) males marked at higher levels (Mean = 193.5, SE = 36.58), as compared to group housed (virgin) males (Mean = 40.1, SE = 15.30, *t*(1, 20) = 3.86, *p* = 0.001). Virgin males marked at significantly higher levels when females are present (Mean = 436.17, SE = 142.83), as compared to the control arena (Mean = 40.1, SE = 15.30, *t*(1, 16) = −3.607, *p* = 0.003). In contrast, bonded males showed no difference in marking between the control arena (Mean = 193.5, SE = 36.58), and one that contained unfamiliar females (Mean = 105.75, SE = 46.47, *t*(1, 14) = 1.34, *p* = 0.20). We found a non-significant trend (due to Bonferroni corrections) for both virgin (Mean = 234.00, SE = 92.359, *t*(1, 18) = −2.315, *p* = 0.034) and bonded (Mean = 383.89, SE = 87.939, *t*(1, 19) = −2.07, *p* = 0.05) males to increase marking in an arena previously marked by a male conspecific as compared to the control arena (Mean = 40.10, SE = 15.304 and Mean = 193.50, SE = 35.582, respectively).

### Overmarking

As predicted, a main effect of species on number of overmarks deposited was found (*F*(1, 105) = 6.503, *p*<0.02) such that *P. californicus* (Mean = 22.94, SE = 5.995) overmarked more than *P. leucopus* (Mean = 6.25, SE = 1.709, t(33) = 2.607, *p* = 0.014), but no significant interaction between species and mating status was found.

Moreover our analysis revealed a significant main effect of species on percent of conspecific donor scent marks overmarked by the focal animal (*F*(1, 105) = 7.957, *p*<0.009). *P. californicus* males (Mean = 0.189, SE = 0.046) overmarked a larger percentage of conspecific marks than did *P. leucopus* males (Mean = 0.043, SE = 0.008), *t*(32) = 2.926, p = 0.006). No significant interaction between species and mating status was found.

### Distribution of scent marks

**Figure 3:**
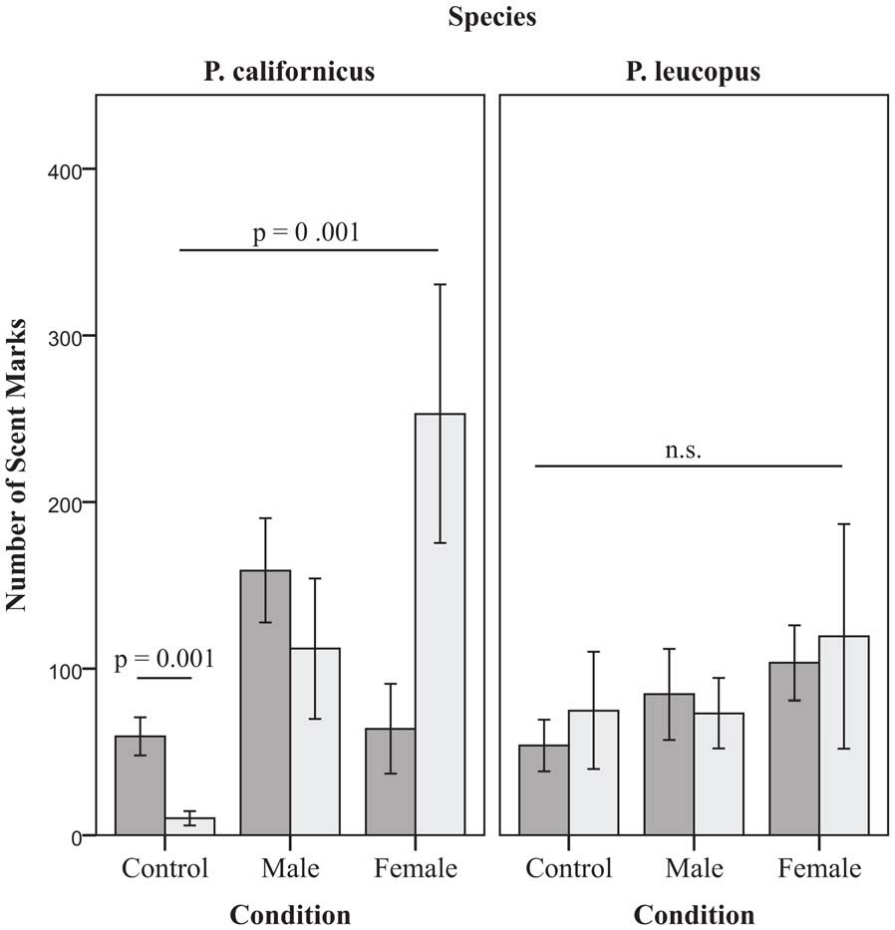
Pair bonding influences total number of scent marks deposited around the perimeter of the arena by focal males (Mean ± SE) in response to male and female stimuli. Dark gray bars represent bonded males and light gray bars represent group housed (virgin) males.

We examined the distribution of scent marks around the perimeter of the arena and found no main effect of species on distribution of urine deposited on the perimeter (*F*(1, 105) = 1.729, *p*<0.2). However, there was a significant three-way interaction (*F*(2, 105) = 3.945, *p*<0.02) between species, mating status and social context (control, male scented, or females present) such that both social context and mating status influence perimeter marking in *P. californicus*, but not in *P. leucopus* (Figure 3). The within-species pairwise comparisons were conducted, as above, using Bonferonni adjusted levels of 0.007 per test (0.05/7). In the control arena, bonded male *P. californicus* (Mean = 59.5, SE = 11.471) deposited more marks around the outer perimeter than virgin males (Mean = 10.2, SE = 4.297, *t*(20) = 4.025, *p* = 0.001). Consistent with total number of marks, virgin males marked around the perimeter at significantly higher levels when females are present (Mean = 253.00, SE = 77.654), as compared to the control arena (Mean = 10.2, SE = 4.297, *t*(1, 16) = −4.117, *p* = 0.001). In contrast, bonded males showed no difference in perimeter marking between the control arena (Mean = 59.5, SE = 11.471), and one that contains novel females (Mean = 64.00, SE = 26.892), *t*(1, 14) = −0.184, *p* = 0.9).

## Discussion

It is widely accepted that mammals scent mark their territories. Several alternative hypotheses about the function of these signals have been proposed, reviewed by [5], [40]. Few studies, however, examine whether this signal can have multiple functions or recipients. Here we examine three non-mutually exclusive, classic hypotheses; demarcation of territorial boundaries, advertisement of competitive ability, and advertisement for potential mates in two closely related species, the highly territorial and monogamous *P. californicus* and the less territorial and promiscuous *P. leucopus*. With classical views in mind, we made several predictions regarding between and within species differences based on our understanding of *Peromyscus* territoriality and mating strategies. We tested previously posed hypotheses and also reveal novel findings regarding the effect of pair bonding on chemical communication.

Scent marking behavior may be expected to evolve when the benefits gained from marking exceed the energetic costs [41] and predation risks [42]–[44] of such behavior. Such benefits may include a reduction in territory intrusions and agonistic encounters if scent marks function to advertise territory ownership and competitive ability, and also reproductive advantage if the function of marking is attracting females (see below). Our findings support previous work indicating plasticity in marking behavior with respect to costs and benefits in *Peromyscus* mice [45] and more generally suggest that scent marks may serve multiple functions that change with pair bond status and level of territoriality.

### Territorial Boundaries and Ownership

Scent marking is most often associated with territory ownership and defense, as scent marks provide physical evidence of dominion and are commonly used by territorial species to delineate territorial boundaries and to deter or intimidate potential conspecific intruders [5], [7], [46]–[48]. Males often defend territories to exclude reproductive rivals and to attract females by controlling access to resources. Further, mating may increase defense, as pair bonding can increase aggression in some species (e.g. prairie voles [49], [50]). In support of the territorial defense hypothesis, we found that both single and bonded male *P. californicus* increased scent-marking around the perimeter of their observation cages in the presence of unfamiliar male scent marks compared to the controls. Also consistent, single male *P. californicus* dramatically increased their perimeter marking behavior in the presence of novel females, whereas bonded males failed to respond to non-mates. Unsurprisingly the less territorial *P. leucopus* did not alter their perimeter marking behavior in response to male stimuli. This finding is consistent with hypotheses that territorial species conduct more perimeter marking than nonterritorial species to delineate their territories. This was found despite the lack of difference in overall frequency of marking between the two species in the control condition.

### Advertisement of Competitive Ability

Males use scent marks, which provide reliable and lasting information about the competitive ability of an individual [4], [9], [51] to advertise identity and presence [52]–[54] as well as assess competitive ability of a territory holder [39]. Socially dominant animals are more likely to scent mark than submissive animals [55], [36]. Further, animals that advertise high competitive ability are less likely to be challenged [56]. Consistent with the advertisement hypothesis, males of the territorial *P. californicus* scent mark and overmark more in response to a same-sex conspecific than the closely related and less territorial *P. leucopus*. *P. leucopus* males may scent mark less, or more randomly, to avoid competitive interactions e.g. [57]–[59] as detection by the first-marker (potential territory owner) will often elicit attacks [60].

### Mate Advertisement

Scent marking behavior may also function as a reproductive strategy to attract mates as females sometimes choose potential mates according to their territorial residence among other things [52], [8], [15]. Evidence from choice studies indicate that females choose mates based on chemical cues found in urine marks [61]. Accordingly, male mice increase their scent marking when they encounter unfamiliar females [62]. In the wild, *P. leucopus* males typically have home ranges that overlap those of several females [32] and mate promiscuously [31], in contrast to *P. californicus* who exhibit territoriality and strict monogamy [17]. Scent marking by males from promiscuous species, and by single males from monogamous species, in the process of searching for a potential mate (e.g. wolves [63]), may be used to advertise to females. Consistent with this idea, when presented with novel female stimuli, single (non-bonded) *P. californicus* males marked more frequently. Bonded *P. californicus* males showed no difference in marking behavior in response to novel females, which suggests that bonded males suppress advertisement to potential mates outside of the pair bond. This is intriguing because it provides further evidence of fidelity in this strictly monogamous species. We found no differences in marking behavior between any of the various social stimuli in *P. leucopus.* Our results therefore provide support for the mate attraction hypothesis, but only in the territorial species.

### Multiple Functions of Scent Marking in Peromyscus

The findings of the current study provide support for several classic hypotheses. No single function appears to account for the marking behavior of *Peromyscus*, likely because scent-marking in a novel environment is functioning differently in territorial versus non-territorial species, and, as found in our study, can serve multiple functions in territorial species. *P. californicus* exhibit plasticity within and between social contexts, in some instances, increasing responses to both male and female stimuli. What is particularly interesting is the influence of mating strategy (strict monogamy) on marking behavior in *P. californicus* males. In essence, pair bonding dampens behavioral responses to novel females, suggesting that bonded males may decrease their allocation of time and energy towards advertising for females in a novel environment. There may also be immediate costs to scent marking, such as attracting predators [41]–[43], [64], resulting in selection against scent marking unless there are benefits outweighing the costs such as attracting females. This effect of pair bonding may be unique to strictly monogamous species or may extend to species that are socially monogamous (pair bonded, but will mate outside the pair).

One caveat to add to our interpretations is whether the results could be explained by a difference in the anxious and fearful responses of the two species because this trait can vary among *Peromyscus* species [65]. As there was no difference between species in surface area covered by marks, it seems unlikely that one species was pooling urine which is typical of stressed individuals such as subordinates [36].

For *P. leucopus,* scent marking does not appear to be directed toward specific male or female stimuli, but rather a general advertisement of presence such as seen in the prairie vole [53]. This does not exclude the possibility that scent marking is used as a form of communication in promiscuous species [44]. There may be something unique that differs in the chemical signals themselves that is not based on frequency [66] or differences may not have been detected in this paradigm. The current findings further elucidate our understanding of the function of scent marking behavior by considering the role of cohabitation with a mate.
